# Orthopedic Surgery Referral Pattern Analysis per Demographic Factors Among Patients Diagnosed With Severe Peripheral Artery Disease in Terms of Partial or Total Limb Amputation

**DOI:** 10.7759/cureus.21455

**Published:** 2022-01-20

**Authors:** Fahad Qureshi, Rachel Amundson, Som P Singh, Prathi Pitchyaiah, Aarya Ramprasad, Serkan Surucu

**Affiliations:** 1 School of Medicine, University of Missouri-Kansas City, Kansas City, USA; 2 Division of Nephrology and Hypertension, The Mayo Clinic, Rochester, USA; 3 Department of Orthopaedics, University of Missouri-Kansas City, Kansas City, USA

**Keywords:** outcome analysis, demographics, quality improvement research, guideline creation, primary care, peripheral artery disease, ai and machine learning, orthopedic surgery referral

## Abstract

Introduction: Peripheral artery disease (PAD) signifies the obstruction of blood vessels in the lower extremities due to harmful buildup of fatty material. Patients may present to their primary care provider complaining of lower extremity pain, especially during exercise. Primary care providers must weigh the severity of patients’ disease process to determine if an orthopedic surgery referral is needed based on an extensive history as well as analysis of demographic factors that may influence their risk of morbidity and mortality. We aimed to objectively present these demographic factors with numeric values in terms of influence.

Methods: We utilized the Cerner Health Facts database to analyze 63 million unique patient encounters from 2000 to 2018. The database is categorized as Institutional Review Board (IRB) exempt due to its de-identified presentation. In an outcome-based approach, we were able to calculate referral patterns based on entered demographic parameters.

Results: A patient’s age, census region, marital status, previous history of PAD/critical limb ischemia (CLI), history of surgeries, race, facility type, and urban/rural status presented as predictors of seeing a surgeon during a patient encounter.

Conclusion: Our results found numerous aforementioned demographic factors to be associated with orthopedic surgery referral patterns. This is significant as proper reconciliation of these factors may help reduce patient morbidity in terms of amputation reduction and reduce patient mortality associated with this surgery or complications.

## Introduction

Peripheral artery disease (PAD) lies under the umbrella of atherosclerotic diseases. Atherosclerotic disease is characterized by plaques that occlude vessels leading to reduced blood flow to the heart, brain, and extremities. PAD occurs when atherosclerosis causes lower extremity arteries to be occluded chronically [1]. The 2016 American Heart Association/American College of Cardiology (AHA/ACC) guidelines state the hallmark of PAD is intermittent claudication, defined by fatigue, discomfort, cramping, or pain of vascular origin in the calf muscle of the lower extremities. Classically, the pain is consistently induced by exercise and consistently relieved within 10 minutes of rest [2]. Patients with this disease suffer from what is colloquially referred to as “poor circulation” [3]. Lack of flow can cause leg pain due to the exertion-induced temporary ischemia. A flow limited vessel is normally defined by a loss of 50% of vessel diameter, associated with a loss in 75% of the cross-sectional area. To ameliorate the lack of blood, flow is shifted to smaller arteries to preserve distal perfusion. However, the agglomeration of small vessels does not carry an equivalent volume of blood, thus rendering the muscles with an inadequate supply of perfusion. Clinically, this presents with difficulty ambulating. While reducing the energy demand of muscle allows for pain relief, when poor perfusion is chronic, tissue can be lost resulting in nonhealing wounds and ischemic ulcers [4].

Critical limb ischemia (CLI) is a grave form of PAD characterized by chronic (>2 weeks) ischemic rest pain, nonhealing wound ulcers, or localized or widespread gangrene [5,6]. It is a common complication with 12% of Americans suffering from the condition [7]. The PAD Awareness, Risk, and Treatment: New Resources for Survival (PARTNERS) study showed that over 70% of primary care providers were incognizant of their patient’s presence of CLI. In particular, the elderly population with CLI also has a high incidence of coexisting coronary artery disease and cerebral vascular disease [8]. Of the 18 million patients diagnosed with PAD, 2 million experience CLI, which increases the risk of losing limbs and general mortality. After presenting with CLI, patients have a 40% rate of needing major amputation surgery and a 20%-25% rate of mortality in the first year alone [9]. CLI is associated with an extremely high risk of adverse events, causing the majority of patients to die of either a cerebrovascular or cardiovascular event [7].

Previous literature has described only racial demographics as related to PAD. African Americans were found to be two to three times more afflicted with PAD as opposed to their white counterparts [10]. It was also found that African American and Hispanic patients suffer from CLI and received major amputations at a greater proportion than their non-Hispanic white PAD patients [11]. 

CLI treatment includes pharmacotherapy, revascularization, and, if necessary, amputation. African American patients and patients with renal disease, PAD, and diabetes mellitus were more likely to receive preamputation arterial testing; however, this subgroup was less likely to obtain a revascularization attempt, insinuating that this decision is due to the extent of disease or other procedural factors. We found that women were also less likely to receive arterial testing, which is congruent with the American Health Association’s statement of women being underdiagnosed for PAD [12].

Hospital resources are utilized extensively by CLI patients. Medicare records in 2014 document CLI patients accounting for 173,000 hospital admissions, 37,000 major lower extremity amputations, and 56,000 inpatient surgical or endovascular revascularizations. In 2013, records show 257,000 hospital-based outpatient cases regarding CLI [13]. In addition, the 2013 to 2014 Nationwide Readmission Database indicated a 20.4% readmission rate in 30 days after an endovascular or surgical intervention on top of this. Based on the Nationwide Readmission Database, the total cost of all hospitalizations for CLI from 2013 to 2014 exceeded 4.2 billion dollars [14]. Medicare reports underestimate the absolute cost of care for CLI patients but still reported a 3.6 billion dollar sum [13]. Hospital length of stay for a patient with PAD is also increased if the patient has comorbidities such as diabetes. Patients who required either amputation or bypass surgery are associated with the highest costs of care [15].

## Materials and methods

**Figure 1:**
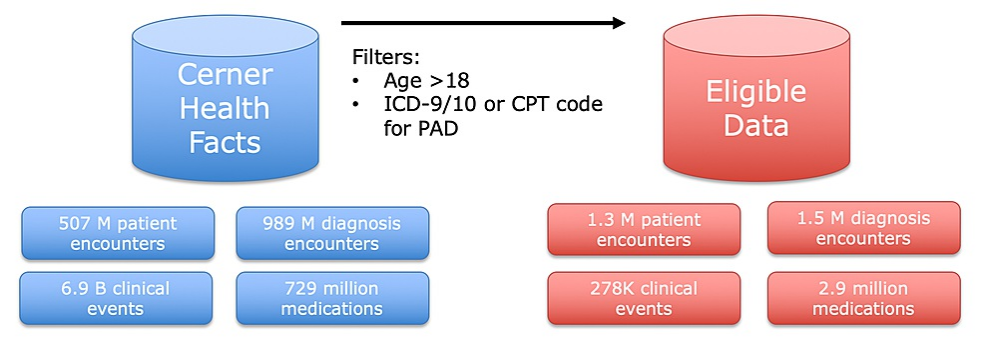
Selection Process From the Cerner Health Facts Database ICD: International Classification of Disease, CPT: Current Procedural Terminology Code, PAD: peripheral artery disease.

As this study was focused on the risk factors predisposing a referral to surgery for an amputation, a retrospective analysis of data was chosen. To ensure an effective number of patients, the Cerner Health Facts database was utilized. The Cerner Health Fact electronic medical record database presented 63 million unique patient encounters from 600 Cerner sites in all 50 states between 2000 and 2018. De-identified patient data were utilized to protect patient privacy, and thus, the research was considered Institutional Review Board (IRB) exempt. Patients were filtered to only those over the age of 18 with a diagnosis of PAD: criteria of which included being marked with an International Classification of Disease (ICD)-9/10 or a Current Procedural Terminology (CPT) code for PAD. Overall, 1.3 million patient encounters were analyzed. Figure 1 visually demonstrates the selection process from the database to the data eligible for our analysis.

Data related to demographics, diagnoses, medications, procedures, and visit site characteristics were identified. To understand the discrepancies in surgeon encounters, these categories were analyzed using logistic regression modeling in Statistical Analysis Software (SAS) by Statistic Solutions to generate odds ratios with 95% confidence intervals. We followed the assumption of independence of observations, limited multicollinearity, linear relation of the logarithm of the odds ratio, and mandated large sample size.

## Results

**Figure 2:**
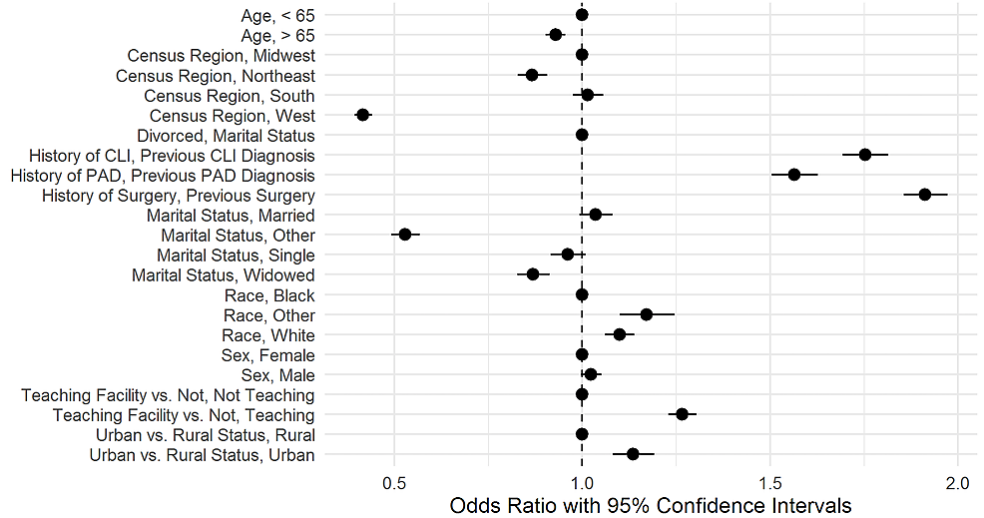
Odds Ratios With 95% Confidence Intervals for Predictors of Seeing a Surgeon During an Encounter After a Peripheral Artery Disease Diagnosis CLI: critical limb ischemia, PAD: peripheral artery disease.

A patient’s age, census region, marital status, previous history of PAD/CLI, history of surgeries, race, facility type, and urban/rural status presented as predictors of seeing a surgeon during a patient encounter. Other variables were not found to be significantly different. Thus, for variables besides the aforementioned ones, we elected not to reject the null hypothesis of a lack of difference. Figure 2 shows the odds ratio with a 95% confidence interval for each of the analyzed factors.

Table 1 demonstrates the demographic factors associated with encounters with surgeons and nonsurgeons.

**Table 1:** Demographic Factor Association With Surgeon and Nonsurgeon Encounters With Attached p-Values In terms of the entries listed in surgeon and nonsurgeon encounters, there are two values listed. The former is the absolute number, and the latter, in parenthesis, is the relative percentage.

Demographic Factor	Category	Surgeon Encounter	Nonsurgeon Encounter	p-Value
Age	<65	113,253 (35.6)	36,471 (39.6)	<0.001
	>65	205,184 (64.4)	55,650 (60.4)	
Census region	Midwest	81,955 (25.6)	16,499 (17.8)	<0.001
	Northeast	52,217 (16.3)	20,704 (22.4)	
	South	157,324 (49.2)	50,098 (54.1)	
	West	28,128 (8.8)	5,265 (5.7)	
Critical limb ischemia		287,935 (90.1)	76,690 (82.8)	<0.001
Peripheral artery disease		63,983 (20.0)	15,773 (17.0)	<0.001
Marital status	Divorced	35,203 (11.0)	10,997 (11.9)	<0.001
	Married	142,883 (44.8)	44,621 (48.3)	
	Other	14,871 (4.7)	3,459 (3.7)	
	Single	59,080 (18.5)	16,834 (18.2)	
	Widowed	67,086 (21.0)	16,419 (17.8)	
Race	Black	57,830 (18.1)	17,824 (19.3)	<0.001
	Other	20,577 (6.4)	4,796 (5.2)	
	White	241,217 (75.5)	69,946 (75.6)	
Sex	Female	149,525 (46.8)	39,843 (43.0)	<0.001
	Male	169,962 (53.2)	52,707 (56.9)	
Facility type	Teaching	85,563 (34.5)	25,565 (29.7)	<0.001
	Not teaching	162,098 (65.5)	60,654 (70.3)	
Urban vs. rural status	Rural	44,519 (13.9)	9,506 (10.3)	<0.001
	Urban	275,105 (86.1)	83,060 (89.7)	

## Discussion

PAD is an extremely common disease that burdens many of our patients, impacting over 8 million in the United States alone [16]. This chronic disease not only causes intermittent claudication, the hallmark of PAD, but also often progresses to a severe form labeled critical limb ischemia. CLI is a severe version of PAD with an increased risk of pain at rest, ulceration, associated complications, and amputations. The literature has shown that amputations are associated with increased morbidity and mortality [17]. We will proceed with certain premises including the need to minimize the need for amputations and therefore the need for early identification and appropriate referrals.

Therapy for CLI includes nonsurgical interventions such as prostaglandin therapy for angiogenesis, wound care, and revascularization. Ideal outcomes have been limited; however, increased quality of life is commonly reported. Amputation is generally viewed as a last resort, once the patient has little anticipation of recovery [18]. The feet and legs generally undergo many abrasions, and stress as an individual is ambulatory and may undergo neuropathy due to the occlusion. Overall, PAD and CLI increase the risk of nonhealing ulcers, and therefore, infection can arise due to these wounds resulting in the need for surgery.

Previous studies have shown non-white patients to be associated with increased major amputation, increased primary amputations, and a lower association of revascularization attempts compared to white patients. Even when adjusting for gangrene and other hospital amputation practices, there was still a significant disparity between non-white and white patients [19]. Our data demonstrated a significant increase in non-white, not including black, referrals to an orthopedic surgeon for amputation consideration compared to black patients. These results were not significant when compared to white patients. Furthermore, our results show white patients being referred at increased rates to surgery when compared to black patients. This warrants further discussion when investigating race in terms of referrals. Considering black patients are afflicted with PAD two- to three-fold when compared to their white counterparts and are receiving lower revascularization attempts, the inconsistent referral rates to surgery are of interest to further study.

A significant disparity in referral rates was found in the type of hospitals patients were seen at. A study using the Veterans Affairs database looked at all major noncardiac operations and discovered that 81% of the total surgical workload and 90% of major surgery workload were performed at teaching hospitals. In addition, patient demographics at these teaching hospitals had an increased prevalence of risk factors and more complex operations on average [20]. While teaching hospitals generally take on more surgical cases, further study of other risk factors is warranted before associating this factor with increased surgery referrals.

Patients who were categorized as a marital status of “other” presented with much lower rates of surgery referral than counterparts who were widowed, single, or married. Marital status has been shown as an independent factor for outcomes in patients who have been hospitalized. In particular, there was an increase in mortality in unmarried patients as opposed to married patients after a surgical procedure. Patients who were unmarried also presented with a higher severity of illness when compared to their married counterparts [21]. The data presented here indicate a lower surgery referral rate for widowed patients when compared to their married cohort. The authors propose that factors that could precipitate this disparity include social support systems, which a physician could consider an unmarried patient to lack and therefore not refer to surgery.

This was a retrospective study analyzing factors that predisposed a surgery referral during an encounter with a PAD patient. Using a database like Cerner is well-suited to a prevalence analysis, such as this study, which allows us to gain insight into associations between factors in a relatively quick and inexpensive manner. However, this data set does not provide insight into factors that clinically influenced physicians as well as patient preferences. Other clinical data could also be limited or missing such as health status and patient characteristics [22]. Coding can also be redundant, where numerous different codes refer to the same prognosis [23]. In this specific study, patients were isolated using an ICD-9/10 or CPT code for peripheral artery disease. However, there may be many patients in the database with this diagnosis who were not coded under this specific code and therefore were not included. Selection bias is also a common challenging issue that affects analysis due to clinical outcomes being influenced by factors that were not recorded in the database such as symptomatology [22]. This bias also comes into play when one considers that only treated patients are documented as opposed to those who may have suffered from autoamputation.

## Conclusions

Peripheral artery disease is the claudication of vessels that results in the ischemia of the peripheral limbs. This ischemia can present in several conditions such as nonhealing wounds, ulcers, neuropathy, pain, and potentially critical limb ischemia. Treatment options include interventions such as wound care, prostaglandins for angiogenesis, and revascularization to salvage limbs. The most drastic intervention remains the gross amputation of the affected limb.

Limitations of this study involved the typical disadvantages of a retrospective study, including a greater propensity to utilize convenience sampling, recall bias, and selection bias. In addition, the lack of corresponding factors that could have influenced the provider’s decision to refer the patient to orthopedic surgery was not analyzed. Finally, this study may not be generalized outside the United States. Strengths of this study include the extensive number of patient encounters; this is the largest patient population analyzed for a study in the literature with only the exception of the government database. The large scale allowed us to increase our power and improve our statistical analysis. In addition, this study is more representative of the United States than any other city or state-wide study, which is common in the literature. Finally, and of note, the literature significantly lacks information regarding the disparate patterns surrounding the referral patterns of PAD, even though the experts that we discussed the project with emphasized the obvious nature of the issue. Thus, this study explores an important yet significantly under-addressed topic.

Several factors influenced the referral rate to orthopedic surgery for said amputation. A patient’s age, census region, marital status, previous history of PAD/CLI, history of surgeries, race, facility type, and urban/rural status were shown to be associated with different referral patterns. These referral patterns are important in our effort to reduce disparity and maximize evidence-based guidelines wherever possible. 
